# Clinical Management of Postoperative Growth Hormone Deficiency in Hypothalamic-Pituitary Tumors

**DOI:** 10.3390/jcm13154307

**Published:** 2024-07-24

**Authors:** Pedro Iglesias

**Affiliations:** 1 Department of Endocrinology and Nutrition, Hospital Universitario Puerta de Hierro, 28222 Majadahonda, Madrid, Spain; piglo65@gmail.com; 2 Instituto de Investigación Sanitaria Puerta de Hierro Segovia de Arana, 28222 Majadahonda, Madrid, Spain

**Keywords:** growth hormone, pituitary tumor, postoperative management

## Abstract

The present review focuses on growth hormone (GH) deficiency in pediatric and adult patients following surgery for hypothalamic-pituitary tumors, with a special emphasis on hormone replacement therapy with recombinant human growth hormone (rhGH). The symptoms and metabolic changes associated with GH deficiency are reviewed, and the potential risks and therapeutic outcomes of rhGH treatment in these patients are discussed. This review emphasizes the importance of rhGH in the normalization of growth in children and the improvement of quality of life (QoL) and metabolic health in adults. Aspects related to efficacy, safety, dosage, duration of treatment, and QoL in this population are analyzed. The need for regular follow-up and dose adjustment to maintain the optimal IGF-I levels in these patients is emphasized, as is the importance of individualized assessment and collaboration with a specialized multidisciplinary medical team to make the appropriate therapeutic decisions. Furthermore, continuous follow-up are necessary to optimize the clinical outcomes in this patient population.

## 1. Introduction

Growth hormone (GH) deficiency (GHD) syndrome is a well-established medical condition resulting from the inadequate production of GH by the somatotrophic cells of the adenohypophysis [1,2]. It can be congenital or acquired, and its etiology varies in children and adults. While in children the cause is usually idiopathic, in adults it is usually secondary to hypothalamic-pituitary tumors and/or their treatment.

The symptoms of GHD may vary depending on the age at the diagnosis of the deficiency. GH plays a key role in growth and development during childhood and adolescence, and therefore its deficiency during this period of life is associated with a reduction in the growth rate, affecting the final height of the individual. However, GH also has important effects i adults, including health-related QoL, exercise tolerance, bone health, and body composition regulation, due to its anabolic and lipolytic effects [3].

The surgical resection of pituitary or other intracranial tumors, with or without radiation therapy, can result in postoperative GHD, with important implications for growth and development in pediatric patients and for metabolic health and QoL in adults. Therefore, the primary goal of hormone replacement therapy with recombinant human growth hormone (rhGH) varies with age. In children, the goal is growth stimulation to normalize the final height, whereas, in adults, the goal is the improvement of QoL and the reversal of metabolic changes associated with GHD [4].

Hormone replacement therapy is the main therapeutic approach for GHD. Replacement therapy with rhGH has been available since the 1980s. Over the years, extensive experience has been gained with its use, and it is widely accepted that it improves or reverses most signs and symptoms of GHD. However, its chronic administration may be accompanied by potential associated risks [5,6].

This review discusses the most relevant aspects of rhGH therapy in pediatric and adult patients with post-surgical GHD in order to facilitate clinical decisions and improve outcomes in this patient population. This information will provide a more complete understanding of the benefits and risks of rhGH therapy, as well as areas for future research to improve the medical care of patients with GHD syndrome after surgery on tumors in the hypothalamus-pituitary area.

## 2. Postoperative Growth Hormone Deficiency

Post-surgical GHD syndrome is a condition characterized by decreased GH secretion following surgery for lesions usually located in the sellar or perisellar area. This deficiency usually occurs as a result of surgery for pituitary tumors or other brain neoplasms, with or without adjuvant radiotherapy [7,8].

In the pediatric population, GHD is idiopathic in 41% of the population, acquired in 35%, and congenital in 20% [7]. Intracranial tumors, alone or in combination with brain radiotherapy, are the major cause (80%) of acquired GHD. These tumors include those distant from the hypothalamus-pituitary area (48.6%), mainly medulloblastoma, astrocytoma, and glioma, and those within the hypothalamus-pituitary area (31.4%), mainly craniopharyngioma, germinoma, and pituitary adenoma. Overall, the tumors of the hypothalamus-pituitary region account for 10.8% of all cases of GH deficiency in the pediatric population [7].

On the contrary, in the adult population, the tumors of the hypothalamus-pituitary region are the most common cause (66.2%) of GHD, mainly pituitary adenoma (53.9%), followed by craniopharyngioma (12.3%) [8]. Within the group of pituitary adenomas, the tumors most frequently associated with post-surgical GHD are, in order of frequency, non-functioning pituitary adenomas (57.7%), prolactinomas (20.5%), somatotroph adenomas (10.3%), corticotroph adenomas (9.0%), and gonadotroph adenomas (2.6%) [9]. 

In patients who have undergone surgery for sellar and parasellar tumors, the combination of low IGF-I levels and at least one additional pituitary hormone deficiency could be indicative of a clear GHD. Consequently, the need for provocative GH secretion testing for the diagnosis of such a deficiency could be avoided [9].

## 3. Consequences of GH Deficiency and Benefits of rhGH Treatment

Table 1 illustrates how GH deficiency can negatively impact growth, development, and overall health, while rhGH treatment can counter these consequences by promoting growth, improving body composition, and enhancing metabolic health.

### 3.1. Growth and Development

GHD is not only the most common pituitary hormone deficiency in children and adolescents, but it is also one of the main causes of short stature in this population [10]. Children with acquired GHD due to intracranial tumors involving the hypothalamus-pituitary region (e.g., craniopharyngioma), with or without cranial irradiation, have severe growth retardation and delayed bone age. Therefore, hormone replacement therapy with rhGH should be considered in this population with persistent GH deficiency following pituitary surgery to promote growth and development and to ensure the desired final height [11,12].

### 3.2. Quality of Life

Patients who develop GHD in both childhood and adulthood often perceive themselves as less healthy and less energetic than normal subjects of the same age [13,14] and have lower scores on QoL questionnaires compared to healthy controls. The treatment with rhGH improves health-related QoL in most patients with GHD, usually during the first year of treatment, although this beneficial effect persists in the medium and long term [15]. This improvement in QoL is most pronounced in women and in patients with poor QoL at the baseline [16,17]. This improvement has also been demonstrated in children with GHD independently of the pharmaceutical form of GH used [18]. 

### 3.3. Body Composition

GHD is associated with changes in body composition, such as an increase in fat mass and a decrease in lean mass [1,2]. Improved body composition is one of the best documented effects of GH treatment in adults with hypopituitarism. A meta-analysis of 22 studies, including 591 GH-treated and 562 placebo-treated adult patients, showed an average increase in lean mass of 2.61 kg in the GH-treated subjects compared with only 0.04 kg in the placebo group. In addition, there was a 2.19 kg reduction in fat mass compared to 0.31 kg in the placebo group. These changes in body composition were dose-dependent [19]. Similar findings have been reported in the pediatric population [20].

### 3.4. Bone Health and Fracture Risk

GHD not only causes stunted growth in children but it can also affect the ability of young adults to reach peak bone mass [21]. This reduction in bone formation and loss of bone mineral density can also increase the risk of osteoporosis. Adult patients with GHD have been found to have increased bone fragility, which increases the risk of fracture by 2 to 7.4 times compared with age-matched controls [22]. Long-term (15 years) rhGH treatment in adults with GHD steadily increases bone mineral content and bone mineral density in the lumbar spine but does not significantly affect the femoral neck [23,24]. Most studies have shown that rhGH therapy in patients with GHD decreases the risk of vertebral and non-vertebral fractures [21].

### 3.5. Liver Function

GH plays an important role in the regulation of liver metabolism and function, particularly in the context of non-alcoholic liver diseases. Different studies have shown that GHD is associated with increased prevalence of non-alcoholic fatty liver disease (NAFLD) and non-alcoholic steatohepatitis (NASH) [25]. The administration of rhGH has been found to reduce hepatic steatosis, inflammation, and fibrosis in overweight/obese individuals with NAFLD, without worsening glycemia [26,27].

### 3.6. Metabolic and Cardiovascular Profiles

Patients with untreated GHD have an increased risk of cardiovascular alterations due to changes in their lipid profile, including elevated levels of total cholesterol, LDL cholesterol, and triglycerides, and decreased levels of HDL cholesterol, as well as insulin resistance and impaired glucose metabolism [28,29]. Elevated levels of pro-inflammatory cytokines such as C-reactive protein (CRP) are also observed. All these changes have been associated with increased cardiovascular morbidity and mortality [29,30,31]. Several studies have shown that hormone replacement therapy with rhGH can positively affect several cardiovascular markers by increasing HDL cholesterol, reducing total and LDL cholesterol, lowering diastolic blood pressure, improving endothelial function, reducing CRP level and carotid intimamedia thickness, and improving cardiac function [32,33,34,35]. Moreover, in adults with GHD, rhGH treatment was associated with a significant beneficial effect on left ventricular mass, interventricular septum thickness, left ventricular posterior wall, and left ventricular end-diastolic diameter and stroke volume as assessed by echocardiography [36]. All of these effects may reduce the risk of cardiovascular disease in GHD patients treated with rhGH in comparison to untreated controls [37,38]. Some studies have also found that middle-aged and elderly patients have similar clinical outcomes, with no significant increase in the risk of rhGH-related adverse events in elderly patients [39].

Acromegaly is a disease associated with metabolic complications, alterations in body composition, and increased cardiovascular risk. A 6-month, randomized, placebo-controlled clinical trial evaluated the effects of GH replacement therapy on body composition and cardiovascular outcomes in patients with GH deficiency following treatment for acromegaly. The results showed a reduction in visceral adipose tissue, an increase in fat-free mass, a decrease in high-sensitivity C-reactive protein levels, and an improvement in quality of life [40].

Hypopituitarism patients with a history of Cushing’s disease tend to have worse metabolic and cardiovascular profiles than hypopituitarism patients following neurosurgery for non-functioning pituitary adenoma. In adults with GH deficiency following Cushing’s disease, treatment with rhGH has also been shown to improve body composition and the cardiovascular risk profile [41]. 

### 3.7. Cardiovascular Disease and Mortality

A number of retrospective and prospective studies have shown that patients with hypopituitarism who receive standard treatment without rhGH have an increased risk of mortality compared to the general population, in particular due to cardiovascular disease [5,42,43]. All-cause mortality and rates of myocardial infarction, stroke, and neoplasia were significantly higher in a group of 1411 hypopituitary patients who did not receive rhGH than in the general population [43]. In this study, where there was an analysis of a cohort of 289 patients diagnosed with hypopituitarism and treated with GH, the rate of myocardial infarction was significantly lower than in the general population, while the neoplasia and mortality rates were similar [43]. 

There are currently no long-term, randomized, controlled trials that have examined the effect of GH replacement therapy on mortality, and they are unlikely to emerge in the future [44]. Therefore, there can be no definitive assurance that GH replacement therapy reduces mortality in patients with GHD. However, several observational studies have shown that the mortality rate of patients with GHD who are treated with GH replacement therapy is lower than that of untreated patients [45,46,47,48]. A meta-analysis showed that the standardized mortality rate in patients treated with rhGH was 1.15 [95% CI: 1.05–1.24] compared to 2.40 [95% CI: 1.46–3.34] in patients not treated with rhGH [46]. However, it cannot be excluded that some selection bias influences the results [48].

## 4. Risks, Side Effects, and Contraindications of rhGH Treatment

The potential risks, side effects, and contraindications of rhGH treatment are summarized in Table 2.

### 4.1. Risk of Tumor Progression and Recurrence and Neoplasia

It is well known that the GH-IGF1 endocrine system plays a key role in growth regulation [49]. Experimental studies showing a permissive role of GH and IGF-I in carcinogenesis have raised concerns about the safety of GH replacement therapy in children and adults who have undergone cancer treatment, as well as in those with intracranial and pituitary tumors (Table 2) [50,51]. 

An international consensus, endorsed by the European Society of Endocrinology and the Growth Hormone Research Society, was published in 2022 to guide decision-making about hormone replacement therapy with rhGH in pediatric and adult cancer survivors, those at increased risk of cancer, and patients treated for intracranial and pituitary tumors [52]. The consensus statement concluded that rhGH does not increase the risk of cancer recurrence or primary tumors in GH-deficient survivors. In addition, its effect on the risk of a second malignancy is less than that of other factors related to the patient and to the treatment of the tumor. There is no evidence of an association between rhGH replacement and an increase in cancer mortality in GH-deficient childhood cancer survivors. The waiting period between completing treatment for an intracranial cancer or tumor and initiating rhGH depends on many factors and should be individualized by the treating physicians, patient, and caregivers. This period can range from 3 months in children with stable craniopharyngiomas to a minimum of 5 years in patients with breast cancer [52].

Recent studies have shown that GH replacement therapy appears to be a relatively safe treatment in terms of tumor progression and recurrence for both children and adults with GH deficiency following surgery for tumors of the sellar/perisellar region, such as craniopharyngiomas and pituitary adenomas, with or without subsequent radiation therapy [53,54,55,56,57,58,59].

#### 4.1.1. Craniopharyngioma

Case-control studies in children and adults with craniopharyngiomas showed no increased risk of tumor recurrence or progression with GH treatment, even in patients with postoperative tumor remnants and in patients treated with or without radiotherapy [53,54,57]. Pediatric patients treated with rhGH after surgical resection of craniopharyngioma had an even lower incidence of tumor recurrence, according to a meta-analysis by Alotaibi et al., 2018 [55]. A systematic review and cohort study published in 2023 that included 18 studies with 6603 patients with childhood-onset craniopharyngioma who were treated with rhGH showed that the treatment was not associated with an increased risk of disease progression, disease recurrence, all-cause mortality, or secondary neoplasms, independent of the timing of the GH replacement therapy [56]. 

#### 4.1.2. Non-Functioning Pituitary Adenoma

The results of several case-control studies have shown that rhGH replacement therapy, aimed at restoring physiologic IGF-1 concentrations, does not appear to have a negative effect on tumor progression or recurrence in patients with surgically treated non-functioning pituitary adenomas [57,59,60,61,62,63]. Although a significant percentage of patients in some of these studies were treated with radiotherapy, which could confound the results, one of the studies that only considered patients with surgery showed similar results in the rate of tumor progression and recurrence between those treated and those not treated with rhGH [61]. Nor has a higher prevalence of malignant neoplasms, both fatal and non-fatal, been observed in adult patients treated with rhGH compared to the general population [63].

#### 4.1.3. Prolactinoma

Macroprolactinomas, and to a lesser extent microprolactinomas, can cause anterior pituitary hormone deficiencies, including GH deficiency. It is currently recommended that patients are assessed for clinical symptoms associated with such hormone deficiencies and treated according to established guidelines [64]. Although the surgical removal of a prolactinoma may resolve hypopituitarism, it may also cause new hormonal deficiencies; therefore, all patients should be reassessed [64]. The potential effect of rhGH treatment on the growth of dopamine agonist-controlled prolactinoma and the possibility of tumor progression or recurrence after surgery is currently unknown.

#### 4.1.4. Acromegaly

The prevalence of GHD syndrome in patients with acromegaly treated with surgery alone or followed by radiotherapy ranges from 2 to 61% [65]. The available information on the effect of hormone replacement therapy with rhGH on GH-secreting somatotropic adenomas after surgery is rather limited. One study of 10 patients with cured acromegaly who received GH replacement for 6 months showed no evidence of an increased risk of the recurrence of pituitary adenomas [66].

### 4.2. Side Effects

The adverse effects associated with rhGH therapy in patients with post-surgical GH deficiency are usually mild and well tolerated (Table 2) [44]. The side effects are most often associated with fluid retention and tend to manifest at the beginning of the treatment or as the dose of rhGH is increased. These effects can usually be alleviated by reducing the dose of rhGH or by discontinuing the treatment [4]. These include injection site discomfort, edema, sensory disturbances, joint pain, and carpal tunnel syndrome [40]. In addition, hyperglycemia or diabetes mellitus may develop as a result of the hyperglycemic action of GH, particularly in obese patients [67]. Elderly patients, patients who are overweight or obese, and those who are over-treated are more likely to experience adverse effects [4,68,69]. 

### 4.3. Contraindications

Because of the possibility that an increase in the IGF-1 axis activity induced by GH treatment may stimulate the growth of malignant tumors, GH treatment is contraindicated in patients with active tumors (Table 2) [4,70]. In patients with tumors of the hypothalamic-pituitary area, treatment with rhGH would be contraindicated in those about to undergo surgery, as well as in those who have undergone surgery with or without subsequent irradiation and where there is suspicion of tumor activity that is not controlled by medical treatment. 

Other contraindications include decompensated or acute cardiovascular disease, severe liver disease, severe renal insufficiency (an estimated glomerular filtration rate of <30 mL/min/1.73 m^2^ body surface area), severe uncontrolled arterial hypertension, active and untreated preproliferative or proliferative retinopathy (diabetic or other), intracranial hypertension, critically ill patients, pregnancy or lactation, and hypersensitivity to GH or any of the excipients of the preparation.

Special caution is recommended when considering hormone replacement therapy with rhGH in patients with an increased risk of malignancies, such as neurofibromatosis type 1, Down syndrome, Bloom syndrome, Fanconi anemia, Noonan syndrome, or DiamondBlackfan anemia [4,71]. 

## 5. Diagnosis of Post-Surgical Growth Hormone Deficiency

**Figure 1:**
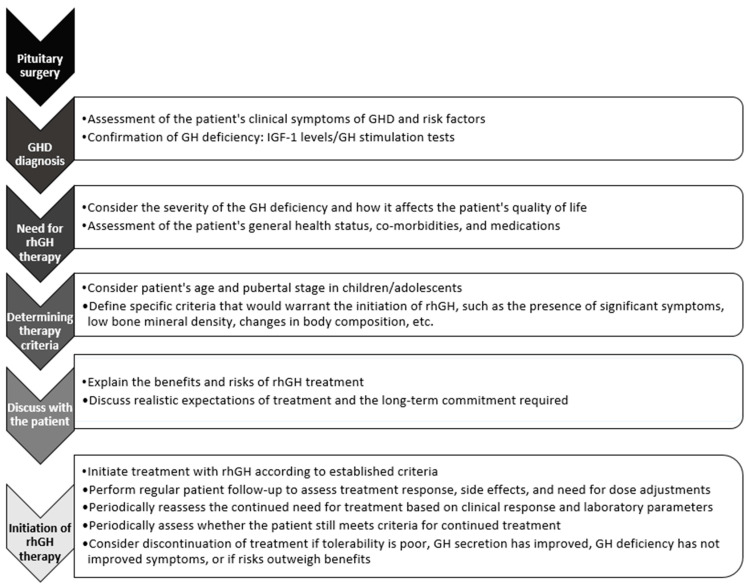
Diagnostic and therapeutic algorithm of post-surgical growth hormone deficiency.

Following the surgical treatment of a tumor in the hypothalamus-pituitary area, with or without radiation therapy, the possibility of developing a GH deficiency syndrome should be considered (Figure 1). This is particularly important in children and adolescents, because the absence of treatment may be associated with growth retardation, affecting the final height of the individual. In adults, it may also adversely affect quality of life; reduce subjective well-being; decrease bone mineral content, muscle strength, and exercise capacity; facilitate changes in lipid profile; and possibly increase cardiovascular mortality. Given the potential risks of tumor re-growth and recurrence, as well as the adverse effects associated with rhGH treatment, a thorough clinical evaluation and appropriate hormonal diagnosis are essential to ensure that the benefits of hormone replacement therapy outweigh the risks in each individual patient.

In a patient with a history of surgery for a hypothalamic-pituitary tumor, it is important to know whether the surgical resection was complete and to have detailed information on the histologic type of the tumor and its proliferation index (Ki-67) before considering diagnostic testing for GHD to initiate hormone replacement therapy. These data are essential because they can have a significant impact on the decision for or against the initiation of GH treatment.

The diagnosis of GHD in childhood includes auxological criteria, bone age, the measurement of serum insulin-like growth factor 1 (IGF-1) and insulin-like growth factor binding protein-3 (IGFBP-3) levels, provocative (stimulation) GH testing, and imaging tests [70,71,72,73]. Most pediatric endocrinologists consider a cutoff point of GH > 10 ng/mL post-stimulus as a normal response; however, with the use of newer assays, the cutoff point has been reduced to 7.5 mcg/L in some countries [70,71,72,73]. Severe GH deficiency is considered when the peak GH is <3 ng/mL after provocative testing, along with a high prior probability of severe GH deficiency based on clinical, laboratory, and imaging information [72]. The major GH stimulation tests used in the diagnosis of GH deficiency include the insulin tolerance test (ITT), the clonidine, the growth hormone releasing hormone (GHRH), the arginine, the glucagon, and the levodopa stimulation tests [70,72]. However, GH secretion stimulation tests are not necessary in children and adolescents when there are clinical, auxological, and hormonal criteria (e.g., low serum IGF-1 and IGF-BP3 levels) that are compatible with GHD; in cases of hypothalamic-pituitary tumors treated with surgery and/or radiotherapy; and in patients with a combined deficiency of at least one other adenohypophyseal hormone secondary to surgery [71].

During the transitional period, from the end of puberty to the time when adult muscle and bone composition and stature are achieved, it is usually necessary to reassess GH secretion prior to initiating GH replacement therapy, because in many cases GHD is transient. For reassessment, GH treatment should be discontinued for at least 1 month and the above stimulus tests should be performed. In this period, a GH response lower than 5 ng/mL after the stimulation provocative tests is diagnostic of GHD [74]. The re-evaluation of GHD during this period would not be necessary in cases where the likelihood of the recovery of GH secretion is low, such as post-surgical severe GHD due to tumor disease or when associated with another pituitary hormone deficiency [70,71,72,73].

Because the manifestations of GHD are non-specific in adults, the evaluation of adult GHD should be reserved for individuals with a high probability of GH deficiency, such as patients with known hypothalamic-pituitary disease. In these patients with normal IGF-I levels, GH stimulation tests should be performed to establish the diagnosis of GHD. The ITT is the only validated test in adults and should be performed in all cases except those in which it is contraindicated (ischemic heart disease, epilepsy, and persons over 60 years of age) [75]. It is necessary to record blood glucose levels to document hypoglycemia induced as a stimulus for GH secretion. As a second option, any other internationally accepted pharmacological stimulus may be used, such as the glucagon stimulation test [76] or the arginineGHRH test [77]. Severe GHD is considered present and therefore amenable to replacement therapy when the serum GH response is less than 3 ng/mL after stimulation provocative tests, such as the ITT or the glucagon stimulation test [76]. A GHD diagnosis is highly likely when the patient has other documented pituitary hormone deficits associated with low serum IGF-1 concentrations. In these cases, it is not necessary to perform GH stimulation tests to establish the diagnosis [78]. In cases of childhood-onset GH deficiency of organic (tumor) origin, the GH deficiency is usually permanent [4,70,71].

## 6. Therapeutic Management: When to Start rhGH Treatment after Surgery and for How Long?

The Endocrine Society recommends the prospective longitudinal growth monitoring of childhood cancer survivors at high risk for adult short stature and the offering of rhGH treatment to these patients with confirmed GHD based on the demonstrated safety and efficacy in this population [79]. It also recommends waiting at least one year disease-free after completion of oncologic treatment before initiating treatment with rhGH. However, the indication to initiate rhGH should always be discussed with the patient’s treating oncologist [79].

In children who have undergone surgery for craniopharyngioma, the initiation of GH treatment may vary depending on the individual circumstances of each patient. It has been suggested that GH replacement therapy could be safely initiated 3–6 months after the last treatment for craniopharyngioma [52,80]. In children with craniopharyngioma and radiologically stable disease and GHD who have significant growth retardation and metabolic disturbances, the initiation of GH therapy may be considered as early as 3 months after treatment [52,56]. A recent single-center, observational, retrospective study found no evidence of an association between the timing (6 or 12 months) of the initiation of GH replacement therapy after treatment for childhood-onset craniopharyngioma and an increased risk of tumor recurrence or progression after complete resection [80]. 

For other types of tumors, it is recommended to wait at least 1 year after the end of the tumor treatment and to start GH only when stability has been confirmed radiologically, considering that tumor recurrence is highest during the first 12 months after the cancer treatment [52]. This period may last up to at least 5 years in adults with a history of solid tumors, such as breast cancer [52]. 

The recommended starting dose of rhGH in children with GHD is 0.16 to 0.24 mg/kg/week (22–35 μg/kg/day) divided into six to seven daily subcutaneous injections with an individualized dose adjustment according to the serum IGF-1 levels. The dose of rhGH should be reduced if the serum IGF-I levels rise above the laboratory-defined normal range for the patient’s age or pubertal stage. It is recommended that the treatment be discontinued if a growth rate of less than 2–2.5 cm/year is achieved. The decision to discontinue pediatric dosing prior to achieving this growth rate should be individualized [71,79].

During the transition from adolescence to adulthood, it is critical to avoid the overdiagnosis of and overtreatment in patients with postoperative GHD. This is accomplished by continuously monitoring the growth velocity and serum IGF-I levels and adjusting the GH dose as needed, focusing on maintaining an optimal balance between the efficacy and safety of the treatment [52]. During the transition, GHD also affects body composition, metabolic profile, and bone mineral density. This is important because one of the main goals of GH replacement therapy with rhGH is to achieve peak bone mass [81]. On the other hand, post-traumatic brain injury (TBI), a common cause of GHD, can have cognitive, behavioral, and physical consequences, emphasizing the importance of diagnosing and treating GHD in pediatric and adolescent patients [82]. The required dose of rhGH during the transition period may vary depending on individual factors, such as gender and concurrent treatments [81]. Women on oral estrogen replacement therapy often require higher doses of rhGH because oral estrogens can attenuate the metabolic effects of the hormone on the hepatic GH receptor, thereby reducing the IGF-1 secretion. The use of transdermal estrogens instead of oral estrogens may help to avoid the attenuation of the effect of GH on the liver, which could affect the dose of GH required. GH replacement therapy increases the conversion of T4 to T3. Therefore, the close monitoring of the thyroid function is important after the initiation of the GH therapy, as it may lead to changes in the levothyroxine dosage or reveal the presence of central hypothyroidism.

The continuation of rHGH treatment from childhood into adulthood has been shown to improve metabolic status, body composition, and bone mineral density in patients with persistent GHD, highlighting the long-term benefits of treatment during the transition period [83]. On the other hand, the discontinuation of GH treatment during the transition period in patients with GHD, starting in childhood, can lead to adverse metabolic changes, highlighting the need to initiate or restart treatment early to avoid adverse health outcomes [84]. The need for close collaboration between the pediatric endocrinologist and the adult endocrinologist is essential for the proper management of these patients.

In adults with post-surgical GHD, rhGH treatment is generally initiated when the deficiency is confirmed and the need for hormone replacement is established [52,78,79]. The decision to start rhGH treatment in these patients should be based on a thorough evaluation of clinical symptoms, hormone levels, the presence or absence of a post-surgical tumor remnant, the histologic type of tumor removed and the degree of cell proliferation, comorbidity, contraindications, and the assessment of the risk-benefit for each individual patient (Table 1 and Table 2) [52,78,85]. 

The starting dose in adults with GHD is generally 0.2–0.4 mg/day s.c. (0.1–0.2 mg/day in those over 60 years of age), with increases of 0.1–0.2 mg/day every 6 weeks until the serum IGF-1 levels are at the upper end of the normal range. Adequate clinical (quality of life, tolerability, and adverse effects) and analytical (blood glucose, HbA1c, lipids, and serum IGF-1 levels to ensure adequate compliance) monitoring is required. After the dose stabilization, follow-up visits may be every 6 months [78,86].

For GHD adult patients with acromegaly after surgery and/or radiotherapy, low-dose rhGH replacement is suggested in the absence of contraindications [78]. These recommendations are based on the evidence that GH replacement may be beneficial in these patients to improve quality of life and body composition, without increasing the risk of glucose intolerance [87]. 

Because no increased risk of tumor recurrence or progression has been demonstrated with GH treatment in children and adults with craniopharyngioma and non-functioning pituitary adenomas, even in patients with postoperative tumor remnants, whether or not treated with radiotherapy, the European Society of Endocrinology does not consider it to be necessary to treat or monitor patients with the remnants of a pituitary tumor or craniopharyngioma receiving GH replacement differently from those not receiving GH [52]. In case of the recurrence of these tumors, the discontinuation of GH and the re-evaluation of the possibility of reintroduction of GH at a later date should be considered, taking into account the specific characteristics of the tumor and the patient [52].

The duration of GH treatment varies depending on several factors, including the underlying medical condition, the response to treatment, and the individual needs of each patient. 

In children with GHD, the treatment usually begins in infancy and continues until the final adult height is reached. The treatment may continue for several years, often until the epiphyses (ends of the bones) close and skeletal growth is complete. In patients with childhood-onset GHD, the GH-IGF-I axis should be reassessed during the transition period 1 month after the GH withdrawal, except in patients with multiple pituitary hormone deficiencies due to hypothalamic tumors, high-dose radiation (>30 Gy), or hypothalamic pituitary surgery [88,89]. In these latter patients, it is not necessary to discontinue GH replacement [52]. 

In adults with GHD, the treatment with GH is long-term and in some cases may be required for life, depending on the individual response to treatment and the specific medical needs of each patient. The regular monitoring of the serum IGF-I levels and the adjustment of the GH dose as needed to maintain these levels within the normal range is recommended. It is important to continue to closely monitor the response to GH treatment, focusing on achieving an appropriate balance between the efficacy and safety of the treatment [52,79].

## 7. Conclusions

In conclusion, the treatment with rhGH in pediatric and adult patients with post-surgical GHD due to hypothalamic-pituitary tumors is accompanied by an improvement in the quality of life, metabolic health, and proper development of these patients. Therapy with rhGH has been shown to be effective in normalizing the final stature in pediatric patients and reversing the metabolic changes associated with GH deficiency in adults. However, it is critical to establish clear criteria for the initiation, follow-up, and eventual discontinuation of treatment, as well as for the ongoing assessment of the clinical and hormonal responses. To ensure an optimal balance between the efficacy and safety of long-term treatment, specialized medical care and regular monitoring are required.

## Figures and Tables

**Table 1:** Consequences of GH deficiency and benefits of rhGH therapy.

Consequences of GH Deficiency	Benefits of rhGH Therapy
Growth and development	
Growth retardation and delayed bone age	Stimulates linear growth in children
Short stature in adulthood	Increases final height in children
Health-related QoL	
Lower score in QoL questionnaires	Greater benefit in patients with low QoL
Body composition	
Increased fat mass	Reduces body fat accumulation
Decreased lean mass	Increases muscle mass
Bone health and fracture risk	
Reduction in bone formation	Increases bone mineral content
Loss of BMD	Increases BMD in the lumbar spine
Increased risk of osteoporosis	Decreases the risk of vertebral and non-vertebral fractures
Liver function	
Increased prevalence of NAFLD and NASH	Reduces hepatic steatosis, inflammation, and fibrosis in overweight/obese individuals with NAFLD
Metabolic and CV profile	
Elevated total and LDL cholesterol	Reduces total and LDL cholesterol
High triglycerides levels	Lowers diastolic blood pressure
Decreased HDL cholesterol	Increases HDL cholesterol
IR and impaired glucose metabolism	Reduces carotid intimamedia thickness
Elevated pro-inflammatory cytokines (CRP)	Reduces CRP level
	Improves endothelial and cardiac function
CV and global mortality	
Increased risk of CV mortality	Reduces incidence rate of myocardial infarction
	Tendency to decrease global mortality

Abbreviations: BMD, bone mineral density; CRP, C-reactive protein; CV, cardiovascular disease; IR, insulin resistance; NAFLD, non-alcoholic fatty liver disease; NASH, non-alcoholic steatohepatitis; and QoL, quality of life.

**Table 2:** Potential risks, side effects, and contraindications of rhGH treatment.

Potential Risks
Concerns about the possibility of tumor progression and recurrence
Potential increased risk of malignancies in certain medical conditions *
Side effects
Fluid retention
Injection site discomfort
Edema
Sensory disturbances
Joint pain
Carpal tunnel syndrome
Hyperglycemia/diabetes mellitus
Contraindications
Active malignancy
Acute decompensated heart failure
Severe liver disease
Severe renal insufficiency
Severe uncontrolled arterial hypertension
Active and untreated preproliferative or proliferative retinopathy (diabetic or other)
Intracranial hypertension
Critically ill patients
Pregnancy or lactation
Hypersensitivity to GH or any of the excipients of the preparation

* Neurofibromatosis type 1, Down syndrome, Bloom syndrome, Fanconi anemia, Noonan syndrome, or DiamondBlackfan anemia.
